# Enhanced Photocatalytic Properties of PET Filaments Coated with Ag-N Co-Doped TiO_2_ Nanoparticles Sensitized with Disperse Blue Dyes

**DOI:** 10.3390/nano10050987

**Published:** 2020-05-21

**Authors:** Hui Zhang, Qi Tang, Qingshan Li, Qingwen Song, Hailiang Wu, Ningtao Mao

**Affiliations:** 1 Research Centre for Functional Textile Materials, School of Textile Science and Engineering, Xi’an Polytechnic University, Xi’an 710048, China; 2017012047@stu.xpu.edu.cn (Q.T.); liqs@xpu.edu.cn (Q.L.); 19921001@xpu.edu.cn (Q.S.); whl@xpu.edu.cn (H.W.); 2 School of Design, University of Leeds, Leeds LS2 9JT, UK; n.mao@leeds.ac.uk

**Keywords:** Ag-N co-doped, TiO_2_ nanoparticles, sensitization, water-insoluble disperse blue dye, photocatalytic activity

## Abstract

In this study, the effects of disperse blue dye-sensitization on the photocatalytic properties of the Ag-N co-doped TiO_2_ nanoparticles loaded on polyethylene terephthalate (PET) filaments are investigated under visible light irradiation. The microstructure and photocatalytic properties of the as-synthesized TiO_2_ nanocomposites, as well as the as-prepared PET filaments, are systematically characterized. The photocatalytic performance of the PET filaments coated with the Ag-N co-doped TiO_2_ nanoparticles sensitized with disperse blue dyes is evaluated via its capacity of photo-degrading methyl orange (MO) dyes under visible light irradiation. It is found that the holes are the predominant reactive radical species and the hydroxyl and superoxide radicals play a subordinate role in the photocatalytic reaction process. The reaction rate constant of the photocatalytic composite filaments is nearly 4.0 times higher than that of the PET filaments loaded solely with TiO_2_ nanoparticles. The resultant photocatalytic composite filaments are evident to be capable of repeatedly photo-degrading MO dyes without losing its photocatalytic activity significantly.

## 1. Introduction

Titanium dioxide (TiO_2_) has been widely exploited as an efficient photocatalytic semiconductor for the photo-oxidation of a vast variety of organic contaminants because of its low cost, low toxicity, abundance, photo-corrosion resistance, thermal and chemical stability and high oxidation ability [1]. Because of its relatively wide band gap and low separation efficiency of photo-generated charge carrier, enormous efforts have been made to improve the photocatalytic activity of TiO_2_ like nonmetal [2] and metal [3] doping, dye sensitization [4], defect engineering [5] and constructing hierarchical structures [6]. For example, it was demonstrated that the transition metal doping resulted in the local impurity states between the valence band and the conduction band of metal doped TiO_2_ and that the localized intermediated states played as “springboard,” which made the electronic transition from valence band to conduction band become easy [7]. The enhanced photocatalytic activity of the C-N co-doped TiO_2_ is attributed to the synergistic effects of C and N doping under visible light irradiation [8].

Especially, it was reported that noble metal nanoparticles possessed an intense surface plasmon resonance (SPR) absorption in the visible light range [9] and acted as electron sinks to store the excess electrons in conduction band [10]. The SPR effect is derived from the collective oscillation of surface free electrons of conducting metals and the Schottky barrier between noble metal and TiO_2_ preventing these transferred electrons from returning to the noble metal nanoparticles [11]. As far as the noble metals doped TiO_2_ concerned, Ag doped TiO_2_ plasmonic photocatalysts have attracted great attention owing to its relatively low price, excellent conductivity, broad SPR absorption and the resultant local electromagnetic fields of Ag [12]. The adsorptional, substitutional and interstitial sites of Ag at surface and subsurface layers of TiO_2_ have been explicated comprehensively in the literature [13]. It was confirmed by spin-polarized density functional theory (DFT) that the substitutional Ag doped at Ti sites introduced Ag 4d states above the valence-band maximum, which might help in shifting visible-light excited electrons to the conduction band of TiO_2_ [14]. The photo-induced electrons excited to the dopant Ag 4d states could be further transferred to the surface states of Ag and subsequently consumed for the reduction of oxygen via the multi-electron reduction, thus resulting in an enhancement in the photocatalytic activity of TiO_2_ [15]. In addition, the oxy radicals were produced from the Ag doped TiO_2_ under visible light illumination and participated in oxidation reactions to decompose organic components [16]. The coating of magnetite (Fe_3_O_4_) and Ag on TiO_2_ is beneficial for the separation of electron-hole pairs and surface wettability of as-prepared TiO_2_/Fe_3_O_4_/Ag nanocomposites [17].

However, Ag doped TiO_2_ nanocomposites are unstable in the photocatalytic processes due to the oxidation of Ag owning plasmonic holes, leading to the deactivation of catalysts [18]. To improve the photocatalytic efficiency and durability of Ag doped TiO_2_ photocatalysts, nonmetal elements were incorporated into the Ag doped TiO_2_ by utilizing the synergistic effect between doped metal and nonmetal elements [19]. For instance, an N-Ag co-doped TiO_2_/C fibrous mat was prepared by using polyacrylonitrile (PAN) as the substrates and tetrabutyl titanate, silver nitrate, urea as the precursor sources of titanium dioxide, silver and nitrogen, respectively [20]. DFT calculation results indicated that N doping introduces isolated N_2p_ states above the valence band maximum which acted as an electron trap to promote the photo-excited electron-hole pairs recombination [21]. For Ag doped TiO_2_, Ag 4d states were introduced above the valence band maximum which resulted in the band gap narrowing [21]. The synergistic co-doping effects of Ag and N with TiO_2_ possessed stable configuration, narrowed band gap and increased visible light absorption [21]. Furthermore, N doping reduced the band gap of TiO_2_, while Ag doping enhanced the separation of photo-excited electron-hole pairs [21]. The In, Ag and N tri-doped anatase TiO_2_ exhibited the enhanced absorption in the visible region [22].

To take full use of TiO_2_ and recycle particulate TiO_2_ from water, the flexible textile materials were employed as the support to deposit Ag doped TiO_2_ by using different techniques, including layer-by-layer spraying [23], electrospun [24], sol-gel and spinning [25], hydrothermal synthesis [26], photochemical reduction [27] and wet-spinning process [28]. A wide range of textile based photocatalysts were developed by coating Ag doped TiO_2_ nanoparticles onto various fiber substrates, including wool [29], glass [30], PAN [31], polyethylene terephthalate (PET) [32], carbon [33], palygorskite [34] and cotton [35], to impart the UV-protective [36], anti-bacteria [37], self-cleaning [38] and pollution removal [39] properties. It was reported that the introduction of polyoxometalate (POM) species could not only improve the light adsorption and redox activity of TiO_2_ but also promote the photochemical stability of the plasmonic TiO_2_ composites because of the relatively narrow band gap and low reversible redox potential of polyoxoanion [40]. The incorporation of Fe_3_O_4_ and Ag nanoparticles nucleated on TiO_2_ surface could boost the electron-hole pair separation and prolong their recombination rate [41]. On the other hand, dye-sensitization of TiO_2_ nanoparticles, as an alternative and promising approach to induce photocatalysis in visible light region, were also studied extensively. A hydrophilic self-cleaning PET fabric loaded with TiO_2_ nanoparticles [42], which was sensitized by porphyrin dyes having different anchoring groups like carboxylic acid, cyanate and pyridine functionalities, were prepared by the deposition and adsorption methods, showing excellent photo-degradation of rhodamine dye. Although the dye-sensitized TiO_2_ photocatalysts possess the advantages of both flexible tuning activity and low cost, the low stability of water soluble dyes limits their practical application since the anchor groups of dyes, such as carboxylic acids, phosphonic acids, cyanoacrylic acids, catechol and sulfonic acids, display low water resistance. To prevent the dyes from dissolution in water, the superhydrophobic silicon coating was applied to the dye-sensitized TiO_2_ by a dip-coating process and simultaneously fixed on PET substrate via the bonding of O to TiO_2_, resulting in the low energy electron excitation of TiO_2_ band gap [43].

In this paper, a new composite photocatalyst composed of PET filaments loaded with Ag-N co-doped TiO_2_ nanoparticles sensitized with water insoluble disperse blue dyes is reported. The synergetic mechanism of metal and nonmetal elements co-doping and dye sensitization on the photocatalytic activities of TiO_2_ nanoparticles under visible light irradiation is investigated. The hydrothermal method is known to be a facile, time-effective and environmentally friendly process for synthesizing doped TiO_2_ well bonded on the surface of polymeric fibers [44]. The reaction is usually carried out in a closed and pressurized (autoclave) system at slightly higher temperature for a period of time, so might be of relatively high cost [45]. The photocatalytic PET filaments are formed by loading with TiO_2_ nanoparticles simultaneously decorated with Ag nanoparticles, doped with N element and sensitized with aqueous insoluble disperse blue 183 dye in a simple one-step hydrothermal process. In comparison with the PET filaments coated with TiO_2_ nanoparticles, the photocatalytic PET filaments coated with Ag nanoparticle decorated, N element doped and dye-sensitized TiO_2_ particles exhibit much enhanced light absorption capacity, efficient separation efficiency of electron-hole pairs and substantial photocatalytic activity in degradation of methyl orange (MO) dye under visible-light irradiation. Moreover, the energy band structure and photocatalytic mechanism of the dye-sensitized PET-Ag-N-TiO_2_ composite photocatalyst are also studied and elucidated. It is expected that the synergic effect of both doping of Ag nanoparticles and sensitization with disperse dyes will lead to a significant improvement of the light harvesting ability of TiO_2_ nanoparticles, as well as their effective separation of photo-generated carriers and good recyclability. The resultant new PET fibrous composite photocatalyst loaded with Ag-N co-doped TiO_2_ nanoparticles could be a new environmentally friendly solution of treating various wastewater from textile, leather and printing industries under visible lights.

## 2. Experimental Section

### 2.1. *Materials and Reagents*

The fully-drawn-yarn (FDY) PET filaments provided by Suzhou Fiber Inspection Institute (Suzhou, China) were used as the substrate. According to the optical projection microscopy method [46], the average diameter of as-received PET filaments was 22.5 ± 0.3 μm by measuring thirty fibers via cutting the filament into short fibers and the corresponding coefficient of variation was 3.32% at 95% confidence level. The average linear density of the filaments was determined as 5.5 dtex based on the volume density of PET 1.38 g/cm^3^. All chemical reagents used in this work were analytical grade, which were procured from Shanghai Aladdin Bio-Chem Technology Co., Ltd. (Shanghai, China), including tetrabutyl titanate (TBT, C_16_H_36_O_4_Ti), silver nitrate (AgNO_3_), tertbutyl alcohol (TBA, C_4_H_10_O), potassium dichromate (K_2_Cr_2_O_7_), ethylenediaminetetraacetic acid (EDTA, C_10_H_16_N_2_O_8_), methyl orange (MO, C_14_H_14_N_3_SO_3_Na, CAS No. 547-58-0), sodium hydroxide (NaOH) and absolute ethanol (C_2_H_6_O). Disperse blue 183 (C_19_H_19_BrN_6_O_3_, CAS No. 2537-62-4) dye was purchased from Shanghai Orgchem Co., Ltd. (Shanghai, China). Distilled water was applied as the solution through the whole experiments.

### 2.2. Simultaneous Dyeing and Depositing Ag–N Co-Doped TiO_2_ Particles on PET Filaments

Prior to treatment, to produce ethylene glycol for reducing AgNO_3_ into Ag nanoparticles [47], the PET filaments were pretreated with 100 g/L of NaOH solution at 100 °C for 45 min to generate some tiny pits on PET filament surfaces on the basis of a material to liquor ratio of 1:80. The filaments were successively washed with absolute ethanol solution at 40 °C and distilled water at 80 °C for 10 min twice until the pH value of the solution was neutral and finally dried at 80 °C in an oven. The mass loss of PET filaments was 10.6% after treatment with alkali solution.

**Scheme 1:**
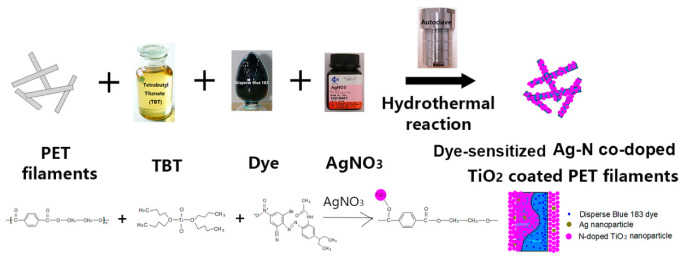
The fabrication route of the dye-sensitized Ag-N co-doped TiO_2_ coated polyethylene terephthalate (PET) filaments (P–2).

Herein, the hydrothermal method was applied for the deposition of Ag-N co-doped TiO_2_ particles on the surfaces of PET filaments, which were simultaneously dyed with disperse blue 183 [48]. The schematic diagram of the preparation route for the dye-sensitized Ag-N co-doped TiO_2_ composite photocatalyst is shown in Scheme 1. Typically, based on the mass ratio of TBT to dye and then to AgNO_3_ 1:10:250, 0.6 mg of TBT was added to 5 mL of absolute ethanol solution under vigorous stirring at room temperature, followed by adding 5 mL of 95% ethanol solution. At the same time, 6 mg of disperse blue 183 dye (0.15 mg/mL) was ultrasonically dissolved into 40 mL of distilled water at 28 kHz and 100 W for 10 min. Next, the dye solution was put into the TBT and ethanol mixture solution and 150 mg of AgNO_3_ was immediately added into the above suspension. The total volume of the precursor solution was controlled at 80 mL by addition the distilled water. According to the liquor ratio of PET filaments to precursor solution 1:120, 0.65 g of pretreated PET filaments was accordingly immersed into the precursor solution for 3 min. The suspension including the filaments was then transferred to a 100 mL polytetrafluoroethylene (PTFE)-lined stainless steel autoclave, which was sealed and placed in a reactor and run at a speed of 6 r/min. Afterwards, the autoclave was raised to 120 °C at a heating rate of 1.5 °C/min. After 3 h hydrothermal reaction, the PET filaments were ultrasonically washed with ethanol solution at 40 °C and distilled water at 80 °C for 10 min thrice respectively and finally dried at 60 °C. For the dye-sensitized N-doped TiO_2_ coated PET filaments, the processing condition was kept the same as the dye-sensitized Ag-N co-doped TiO_2_ coated PET filaments except AgNO_3_ was not used. The dye-sensitized N-doped TiO_2_ coated and dye-sensitized Ag-N co-doped TiO_2_ coated PET filaments were denoted as the P–1 and P–2, respectively. Besides, the particles left in the suspension were collected by centrifugation, filtration, washing with anhydrous ethanol and distilled water and drying in a vacuum oven at 60 °C overnight. In addition, the TiO_2_ coated and Ag-doped TiO_2_ coated PET filaments were also fabricated by using TBT and TBT combined with AgNO_3_ according to the above mentioned method without adding of disperse blue 183 dye, respectively. The Ag-N co-doped TiO_2_ coated PET filaments without any dye sensitization were not prepared in this study because the doped N element was derived from the Disperse Blue 183 dye.

### 2.3. Characterization Techniques

The surface morphologies of the pristine, P–1, P–2, TiO_2_ coated and Ag-doped TiO_2_ coated filaments were observed by field emission scanning electron microscopy (FESEM, JEOL JSM–6700F, Tokyo, Japan). Prior to observation, the specimens were mounted on a holder and coated by a layer of sputtered platinum using a vacuum evaporator (JEE-420, JEOL, Tokyo, Japan). The elemental analysis was identified using an INCA Energy 400 energy dispersive X-ray (EDX) spectrometer (Oxford Inst., Oxford, UK) attached to the field emission scanning electron microscope (FESEM). The filament specimens made a platelet with a diameter of 8 mm and a thickness of 1 mm for element analysis.

The crystal phase of as-synthesized particles was analyzed using the 7000S X-ray diffractometer (Shimadzu, Tokyo, Japan) with Cu Kα_1_ radiation (λ = 0.154056 nm) at a scanning rate of 8°/min in the 2θ range of 10–80°. The crystallite size was calculated by using the Scherrer Equation (1) [49].

*D* = K*λ*/(*B*cos*θ*), (1)

where *D* is the crystallite size, K is the crystallite-shape factor which normally takes a value 0.89, *λ* is the wavelength of the X-rays, *B* is the full-width at half-maximum (FWMH) of the X-ray diffraction peak in radians and *θ* is the Bragg angle.

The microstructure of the as-synthesized particles obtained from P–1 and P–2 was further investigated using a transmission electron microscope (TEM, JEOL JEM-3010, Tokyo, Japan) with Digitalgraph software at an accelerating voltage of 200 kV.

The chemical states of the P–1, P–2, TiO_2_ coated and Ag-doped TiO_2_ coated filaments were studied by X-ray photoelectron spectroscopy (XPS) using the Axis Ultra electron spectrometer (Kratos Analytical Ltd., Manchester, UK) with a monochromatic Al K*α* (1486.68 eV) radiation (10 kV, 10 mA) with the vacuum less than 1.33 × 10^−6^ Pa. The binding energy was verified by using the C_1s_ hydrocarbon peak at 284.8 eV.

The measurements of photocurrent-time (*i*–*t*) curves, electrochemical impedance spectroscopies (EIS) and Mott–Schottky (M–S) curves for the P–1 and P–2 filaments were performed on a CHI760E (Shanghai Chenhua Instrument Co., Ltd., Shanghai, China) electrochemical workstation under a 300 W Xe lamp (420–780 nm waveband) irradiation. The standard three-electrode system was adopted by using a Pt foil as the counter electrode and a saturated calomel electrode (SCE) as the reference electrode. The working electrode was fabricated by warping 12 mg of the filaments specimens around the indium-tin oxide (ITO) glass with a size of 1 cm × 1 cm. The applied potential for photocurrent measurement was 0.5 V vs SCE using Na_2_SO_4_ (0.5 M, pH = 7) aqueous solution as the electrolyte. The frequency for EIS measurement was from 1 to 1,000,000 Hz at 5 mV AC voltage amplitude under open circuit potential condition. The potential for Mott–Schottky measurement was from −1 to +1 V at a scanning rate of 5 mV/s and the frequency was 1 kHz.

The steady-state and time-resolved photoluminescence (PL) spectra of the P–1 and P–2 filaments were acquired by a FS5 fluorescence spectrophotometer (Edinburgh Instruments Ltd., Edinburgh, UK) using a Xe lamp as excitation source at an excitation wavelength of 340 nm.

The UV-Vis diffuse reflectance spectra of the PET filaments were obtained by using a Lambda 950 (PerkinElmer, Waltham, MA, USA) spectrophotometer with 150 mm integrating sphere in 200–800 nm range. The barium sulfate pellet was used to calibrate the 100% reflectance.

The valence band (VB) spectra of the P–1 and P–2 filaments were recorded on an Escalab 250 Xi UV photoelectron spectrometer (Thermo Fisher Scientific Inc. Waltham, MA, USA) with a He lamp that had two resonance lines (He I, 21.2 eV and He II, 40.8 eV). The filaments were etched by argon ion before testing.

The electron spin resonance (ESR) measurements of the P–2 filaments were conducted at ambient temperature on a Bruker A 300 apparatus using 5,5-dimethyl-1-pyrroline n-oxide (DMPO) and 2,2,6,6-tetramethylpiperidine-1-oxyl (TEMPO) as the spin trapping reagents for the detection of both hydroxyl (·OH) in water and superoxide (·O_2_^−^) in methanol dispersions [50] as well as for characterization of holes (h^+^) in water dispersion [51], respectively. A 300W Xenon lamp was employed as the light source and a visible light (400–780 nm) bandpass filter was applied to shield the UV rays. The apparatus was operated in 9.85 GHz with a modulation frequency of 100 kHz. The microwave power was 19.3 mW and the modulation amplitude was 1.0 GHz. The absorption peaks were assigned to different radical species based on comparisons of their ESR. The magnetic field was calibrated using 1,1-diphenyl-2-picrylhydrazyl as a calibrant positioned close to the sample.

### 2.4. Evaluation of the Photocatalytic Properties

The photocatalytic properties of the TiO_2_ coated, Ag-doped TiO_2_ coated, P–1 and P–2 filaments were evaluated by the photo-degradation of methyl orange (MO) dye aqueous solutions using a 30 W LED lamp as the simulated visible light. The luminous intensity was measured to be 45,000 Lux by a TES–1332A digital light meter (TES Electrical Electronic Corp., Taiwan, China). In a typical experiment, 0.5 g of the filament samples was added into 50 mL of 2 mg/L MO aqueous solution at neutral pH condition. After 30 min in the dark to attain the adsorption-desorption equilibrium, the dye solution was exposed to visible light irradiation for 180 min. At an interval of 20 min, about 3 mL solution was abstracted from the solution and the absorbance, *A*, was measured using a VIS–7220 spectrometer (Beijing Rayleigh Analytical Instruments Co., Ltd., Beijing, China) at the maximum absorption wavelength of 464 nm. The concentration of *C_t_* was calculated based on the calibration curve of MO dye solution (*A_t_* = 0.0359 + 0.0678*C_t_*, *R*^2^ = 0.99). The degradation efficiency *E* (%) of the MO dye was calculated by Equation (2):

*E* = (1 − *C_t_*/*C*_0_) × 100%, (2)

where *C*_0_ and *C_t_* were the concentrations of the dye solution at initial time and at *t* time, respectively. The average degradation efficiency was given after three measurements. Furthermore, the reusability of the P–2 filaments was successively measured for 5 cycles by replacing the oxidized MO dye solution with the fresh MO solution under the same visible light irradiation condition.

The roles of various reactive radical species, including hole (h^+^), hydroxyl radical (•OH) and superoxide radical (•O_2_^−^) generated during the photocatalytic process, for the P–2 filaments were assessed by adding the scavenger agents, 1 mM EDTA [52], 0.5 mL TBA [53] and 0.5 mM potassium dichromate [54] to impair h^+^, •OH and •O_2_^−^ to the solution, respectively.

## 3. Results and Discussion

### 3.1. Morphologies, Compositions and Structures

**Figure 1:**
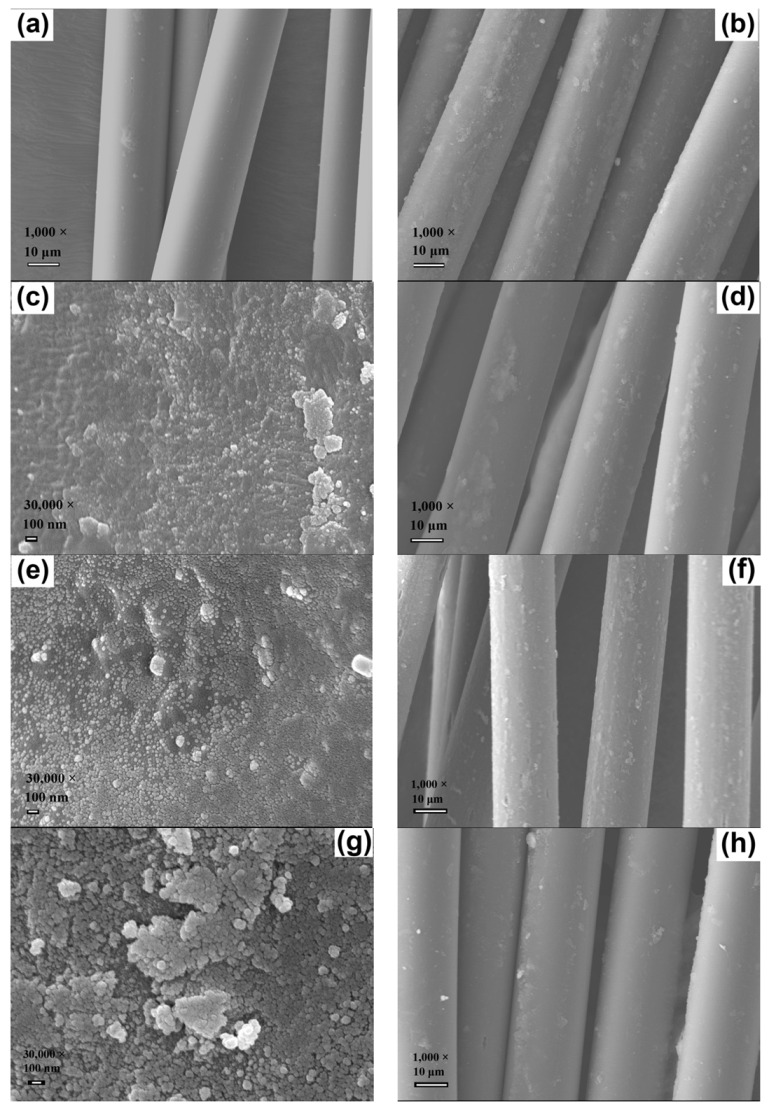

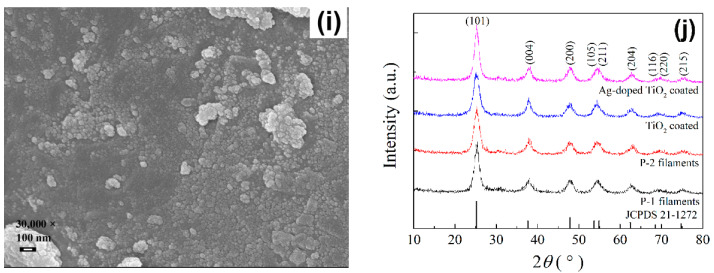
The field emission scanning electron microscope (FESEM) images for the (**a**) pristine filaments, 1000×; (**b**) P–1 filaments, 1000×; (**c**) P–1 filaments, 30,000×; (**d**) P–2 filaments, 1000× and (**e**) P–2 filaments, 30,000×; (**f**) TiO_2_ coated filaments, 1000× and (**g**) TiO_2_ coated filaments, 30,000×; (**h**) Ag-doped TiO_2_ coated filaments, 1000× and (**i**) Ag-doped TiO_2_ coated filaments, 30,000×; (**j**) X-ray diffraction (XRD) patterns of the as-synthesized TiO_2_ particles.

**Figure 2:**
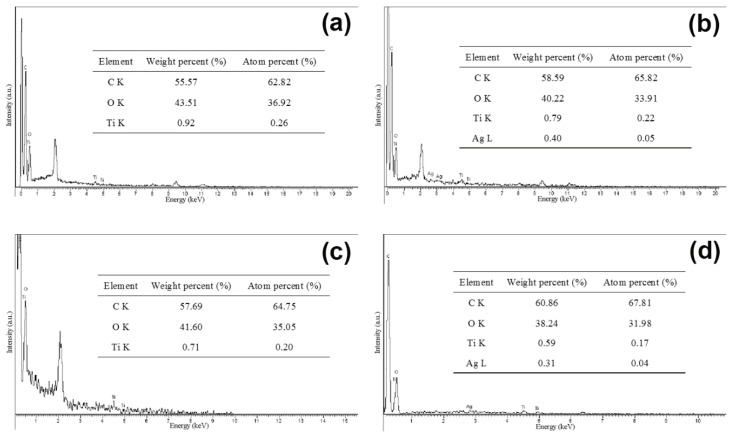
The energy-dispersive X-ray spedctroscopy (EDS) spectra of the (**a**) P–1, (**b**) P–2, (**c**) TiO_2_ coated and (**d**) Ag-doped TiO_2_ coated PET filaments.

The FESEM images of the surface morphologies for the pristine, P–1, P–2, TiO_2_ coated and Ag-doped TiO_2_ coated PET filaments are displayed in Figure 1a–i. It is seen that the surface of the pristine filaments is very clean and smooth without any foreign substances (Figure 1a). After the hydrothermal treatments, the surfaces for both P–1 and P–2 filaments are totally covered by a layer of particulate matter. There is no distinct difference between the P–1 (Figure 1b) and P–2 (Figure 1d) filaments in appearance. From the high-resolution FESEM images, these macro-sized particles are composed of nano-scaled particles, which are uniformly loaded on filament surfaces (Figure 1c,e). Moreover, some nanoparticles are agglomerated to form the sub-micrometer sized granules scattering by random on both filament surfaces. For the filaments coated with TiO_2_ and Ag-doped TiO_2_ nanoparticles, there is no significant change in appearance (Figure 1f,h). The particle sizes measured from the high-resolution FESEM images (Figure 1g,i) are slightly reduced after the disperse blue dye is presented in the TiO_2_ nanoparticles. This is because the dye molecules play a role of structure-directing agent in controlling the growth of TiO_2_ nanoparticles and this is in line with the previous results [48].

The crystal structures of the as-synthesized particles are analyzed by X-ray diffraction (XRD) powder diffraction technique, as depicted in Figure 1j. It is apparent that the diffraction peaks for all the as-prepared particles are indexed to the typical anatase TiO_2_ (JCPDS No. 21-1272) [55] without any other crystalline phases, suggesting the high purity of the as-synthesized TiO_2_. The diffraction peaks centered at 2θ = 25.3°, 36.9°, 48.0°, 53.9°, 55.0° and 62.1° are in accordance with the (101), (004), (200), (105), (211) and (204) crystal planes of anatase TiO_2_, respectively. However, the characteristic peaks of Ag crystalized particles in both P–2 and Ag-doped TiO_2_ coated onto the PET filaments were not detected, this might be because the content of Ag element is too little and out of the sensitivity capacity of the instrument used here. The average crystallite sizes of the as-synthesized TiO_2_ particles are calculated to be 10.7 nm for the P–1 filaments, 11.5 nm for the P–2 filaments, 7.7 nm for the TiO_2_ coated filaments and 6.7 nm for the Ag-doped TiO_2_ coated filaments by using the Scherrer equation based on the (101), (004) and (200) planes of TiO_2_.

The energy-dispersive X-ray spedctroscopy (EDS) spectra together with chemical compositions of the P–1, P–2, PET filaments coated with TiO_2_ nanoparticles and PET filaments coated with Ag-doped nanoparticles are shown in Figure 2. The Ag element is found not only in the P–2 filaments but also in the filaments coated with Ag-doped TiO_2_ nanoparticles. When the disperse blue dye is applied to TiO_2_ nanoparticles, the atomic percent of Ti element in the filaments coated with TiO_2_ increases from 0.20% to 0.26% in the P–1 filaments. Also, both Ti and Ag elements by atomic percent increase from 0.17% and 0.04% for the filaments coated with Ag-doped TiO_2_ to 0.22% and 0.05% for the P–2 filaments, respectively. Thus, the addition of disperse dyes into TBT reaction solution during the hydrothermal process might favor the deposition of TiO_2_ or Ag-doped TiO_2_ particles on filaments. However, the N element is not detected in both P–1 and P–2 filaments because the content of N is beyond the detection limit of the instrument.

**Figure 3:**
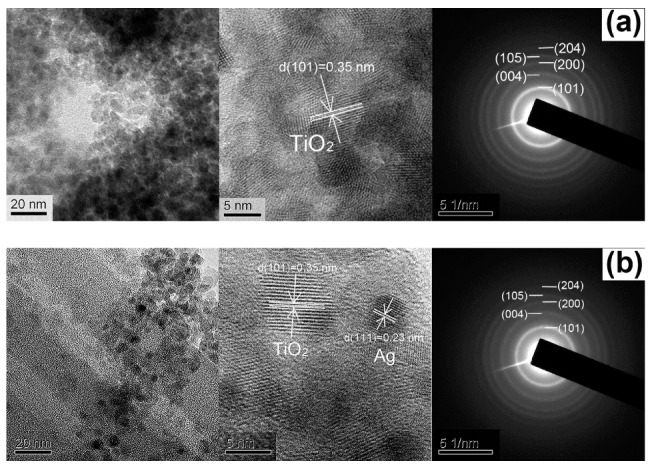
The transmission electron microscopy (TEM) and high resolution TEM (HRTEM) images and selected area electron diffraction (SAED) patterns of as-synthesized TiO_2_ nanoparticles obtained from the (**a**) P–1 and (**b**) P–2 filaments.

The microstructures of as-synthesized TiO_2_ particles exfoliated from both P–1 and P–2 filaments are further examined by TEM analysis. The TEM and high-resolution TEM (HRTEM) images as well as selected area electron diffraction (SAED) patterns are shown in Figure 3. It is evident that the spherical-like TiO_2_ nanoparticles are grown on the P–1 and P–2 filaments with an average diameter of about 5 nm, indicating the particle sizes of TiO_2_ are not restrained by the introduction of organic dye. The inter-planar distances for both filaments are measured to be 0.35 nm in the HRTEM images of TiO_2_ nanoparticles. The results are consistent with the lattice spacing of the (101) plane of anatase TiO_2_ [56]. Importantly, an additional lattice fringe of 0.23 nm corresponding to the (111) plane of Ag [56] is observed in the HRTEM image of TiO_2_ nanoparticles obtained from the P–2 filaments, implying Ag^+^ ions are reduced to Ag^0^ and the Ag nanoparticles are successfully grown and embedded among TiO_2_ nanoparticles. Regarding the SAED images, the rings for both filaments are assigned to the (101), (004), (200), (105) and (204) planes of anatase TiO_2_ [57]. However, the spots of Ag are not found in the corresponding SAED patterns due to the low doping amount of AgNO_3_.

### 3.2. Chemical States

**Figure 4:**
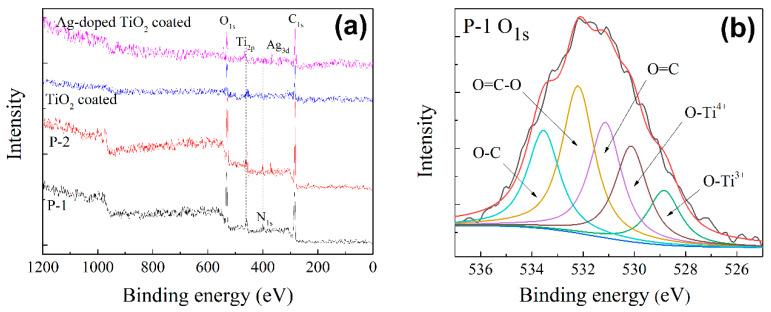

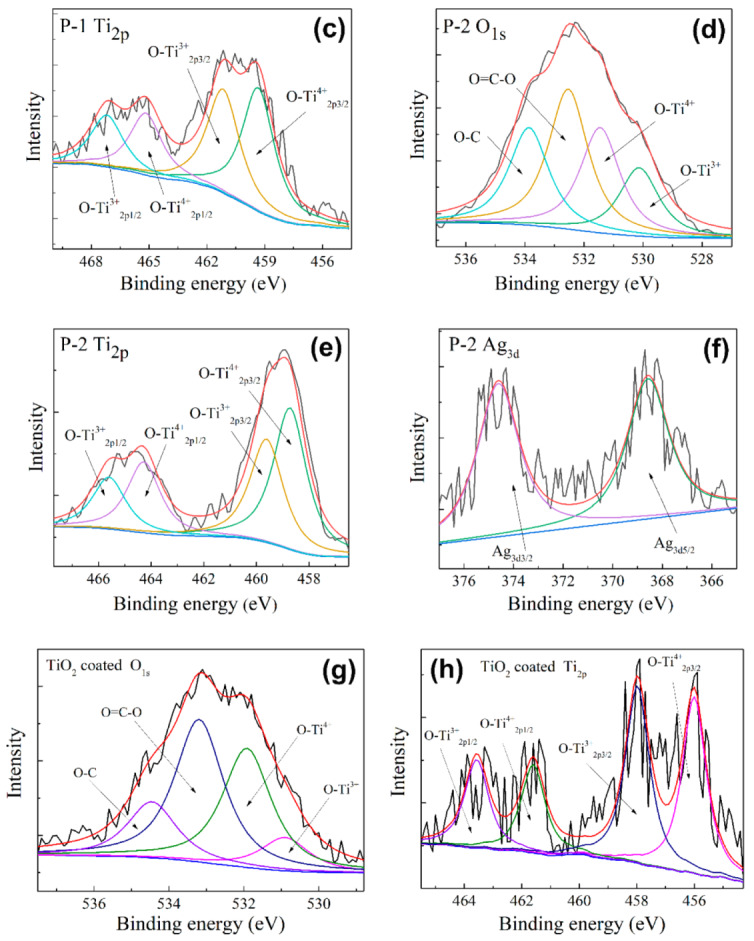

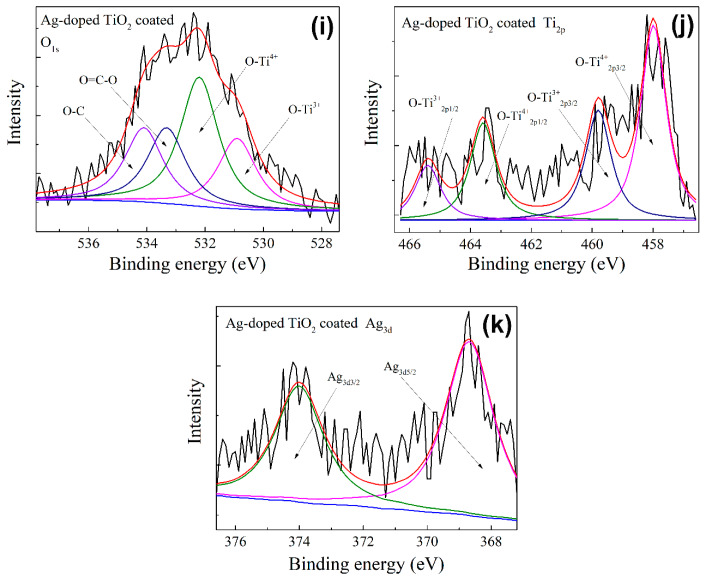
The (**a**) survey spectra for the P–1, P–2, TiO_2_ coated and Ag-doped TiO_2_ coated filaments, (**b**) O_1s_ and (**c**) Ti_2p_ core-level deconvolution spectra of the P–1 filaments, (**d**) O_1s_, (**e**) Ti_2p_ and (**f**) Ag_3d_ core-level deconvolution spectra of the P–2 filaments; (**g**) O_1s_ and (**h**) Ti_2p_ core-level deconvolution spectra of the TiO_2_ coated filaments, (**i**) O_1s_, (**j**) Ti_2p_ and (**k**) Ag_3d_ core-level deconvolution spectra of the Ag-doped TiO_2_ coated filaments

The XPS analysis was employed to elucidate the chemical states of the P–1, P–2, TiO_2_ coated and Ag-doped TiO_2_ coated PET filaments, as illustrated in Figure 4. The quantitative elemental analysis results are summarized in Table 1. The N element is found in both P–1 and P–2 filaments, indicating that anatase TiO_2_ nanoparticles are doped with the element of N, which is caused by disperse blue 183 dye. As expected, the element N is absent from the filaments coated with TiO_2_ and Ag-doped TiO_2_ nanoparticles respectively. The element Ag is detected in both P–2 and the filaments coated with Ag-doped TiO_2_ nanoparticles due to the usage of AgNO_3_, while there is no Ag element detected in both P–1 and the TiO_2_ coated filaments.

By comparison of P–1 and P–2, besides the elements of C, O, N and Ti, the Ag element is identified in the survey spectrum of the P–2 filaments (Figure 4a) and the corresponding atomic concentration is 0.12%. The Ti atomic concentration of the P–2 filaments is 2.49%, higher than 1.81% of the P–1 filaments. The O_1s_ signal of the P–1 filaments (Figure 4b) can be deconvoluted into five sub-peaks of O-C (533.5 eV), O=C-O (532.2 eV), O=C (531.1 eV), O-Ti^4+^ (530.1 eV) and O-Ti^3+^ (528.8 eV). However, the O_1s_ XPS spectrum of the P–2 filaments (Figure 4d) is fitted to four sub-peaks of O-C (533.9 eV), O=C-O (532.5 eV), O-Ti^4+^ (531.5 eV) and O-Ti^3+^ (530.1 eV). Noticeably, the binding energy of O=C is disappeared. Moreover, the O-Ti^4+^ and O-Ti^3+^ bonds of the P–2 filaments are shifted to higher binding energy compared to those of the P–1 filaments. This is because the electron migration transferring from TiO_2_ to Ag. It has been demonstrated that Ag can accept electrons, meanwhile the electrons migrate to Ti^4+^ to form Ti^3+^ (Ag + e^-^ → Ag^-^, e^-^ + Ti^4+^ → Ti^3+^) [58]. The high-resolution core-level Ti_2p_ spectra confirm the coexistences of Ti^4+^ and Ti^3+^ in TiO_2_ for both filaments. For the P–1 filaments (Figure 4c), the sub-peaks centered at 457.2 eV and 463.0 eV (spin orbit splitting of 5.8 eV) are assigned to Ti^4+^_2p3/2_ and Ti^4+^_2p1/2_, while the sub-peaks located at 459.0 eV and 465.0 eV are attributed to Ti^3+^_2p3/2_ and Ti^3+^_2p1/2_, respectively [58]. In the case of the P–2 filaments (Figure 4e), the sub-peaks at 458.7 eV and 464.3 eV (spin orbit splitting of 5.6 eV) are associated with Ti^4+^_2p3/2_ and Ti^4+^_2p1/2_ and the peaks at 459.6 eV and 465.6 eV are ascribed to Ti^3+^_2p3/2_ and Ti^3+^_2p1/2_, respectively. In addition, two characteristic sub-peaks of Ag_3d3/2_ and Ag_3d5/2_ are found at 374.6 eV and 368.6 eV (Figure 4f), respectively. The results indicate that Ag is existed in the form of Ag^0^ in the P–2 filaments [58]. As a result, the Ag-N co-doped TiO_2_ would have a decreased electron density. An internal electric field can be formed in the interface between TiO_2_ and Ag nanoparticles [59].

The O_1s_ signal of the TiO_2_ coated filaments (Figure 4g) is deconvoluted into four sub-peaks of O-C (534.5 eV), O=C-O (533.2 eV), O-Ti^4+^ (531.8 eV) and O-Ti^3+^ (531.0 eV). The corresponding Ti_2p_ signal (Figure 4h) is fitted to four sub-peaks of O-Ti^4+^_2p3/2_ (455.9 eV), O-T^4+^_2p1/2_ (461.5 eV), O-Ti^3+^_2p3/2_ (457.9 eV) and O-Ti^3+^_2p1/2_ (463.5 eV). The O_1s_ signal of the Ag-doped TiO_2_ coated filaments (Figure 4i) is also deconvoluted into four sub-peaks of O-C (534.1 eV), O=C-O (533.3 eV), O-Ti^4+^ (532.1 eV) and O-Ti^3+^ (530.8 eV) and the corresponding Ti_2p_ signal (Figure 4(j)) is fitted to four sub-peaks of O-Ti^4+^_2p3/2_ (457.9 eV), O-T^4+^_2p1/2_ (463.5 eV), O-Ti^3+^_2p3/2_ (459.9 eV) and O-Ti^3+^_2p1/2_ (465.5 eV). Moreover, the Ag_3d_ signal of the Ag-doped TiO_2_ coated filaments is deconvoluted into two sub-peaks of Ag_3d5/2_ (368.6 eV) and Ag_3d3/2_ (373.9 eV), suggesting the existence of the metallic Ag phase [58]. In comparison with the TiO_2_ coated filaments, the increases of the binding energies of O-Ti^4+^ and O-Ti^3+^ for the Ag-doped TiO_2_ coated filaments are due to the interaction of Ag^+^ ions with TiO_2_ [59].

### 3.3. Separation Efficiency of Photo-Generated Electron-Hole Pairs

**Figure 5:**
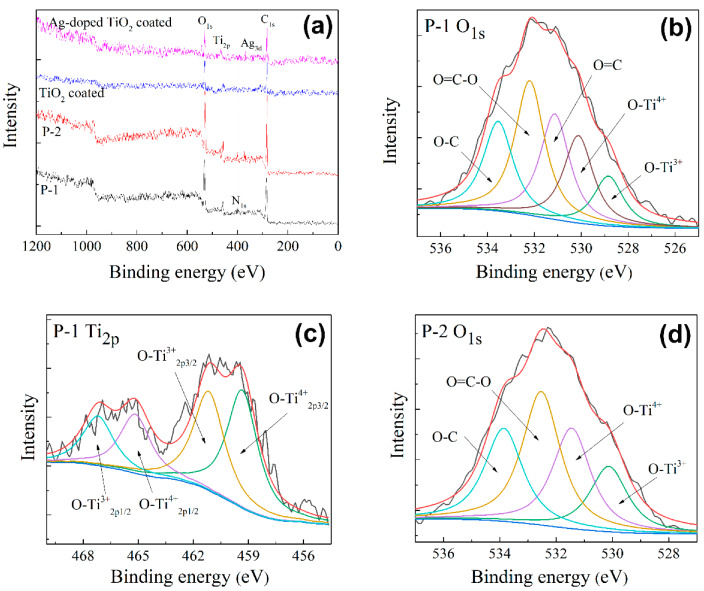
(**a**) Photocurrent response (*i*–*t*) curves, (**b**) electrochemical impedance spectroscopy (EIS) Nyquist plots, (**c**) steady-state PL spectra and (**d**) time-resolved transient photoluminescence (PL) decay spectra of the P–1 and P–2 filaments (λ(E_x_) =340 nm).

The separation efficiencies of photo-generated electron-hole pairs of the P–1 and P–2 filaments are compared by using *i*–*t* curves, EIS Nyquist plots, steady-state PL and time-resolved transient PL spectra and the corresponding results are represented in Figure 5. As described in Figure 5a, in comparison with the P–1 filaments, the P–2 filaments possess the larger photocurrent density in the *i*–*t* curves, meaning the enhanced separation and migration efficiency of photo-generated electron-hole pairs [60]. This is because the doped Ag nanoparticles can easily attract the electrons. The fast electron transfer process not only separates the electrons and holes quickly but also reduces the electron-hole recombination rate and results in an obvious increase of free carrier density in the P–2 filaments [61]. Additionally, the photocurrent densities slightly decrease with the increases of switch-on/off cycles. It means that the recombination of electron-hole pairs occurs in both filaments during the cycle process [61]. When the light source is stitched off (light off), the free carriers disappear slowly in the P–2 filaments because some free carriers are trapped in the Ag nanoparticles, thus there is a delay for the photocurrent in the P–2 filaments.

As stated in Figure 5b, both filaments exhibit the semi-circle at the high and middle frequency region and a straight line at the low-frequency region in the EIS Nyquist plots. This suggests that the electrochemical reaction is determined by the mix process of charge transfer and diffusion [62]. The arc radius of the P–2 filaments is smaller than that of the P–1 filaments at the high frequency region, implying the effective separation of electron-hole pairs and efficient interfacial charge transfer [63]. The introducing of Ag nanoparticles can reduce the electronic resistance, promote the charge transfer to the surface and thereby improve the photocatalytic activity of the Ag-N co-doped TiO_2_ nanoparticles.

**Figure 6:**
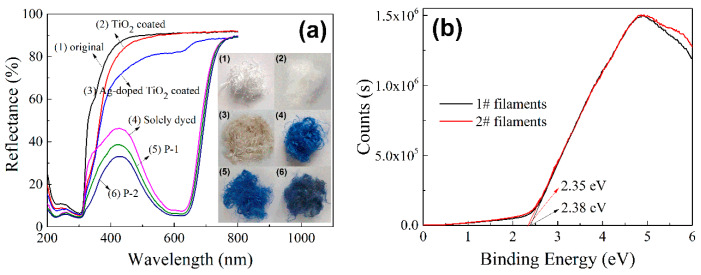

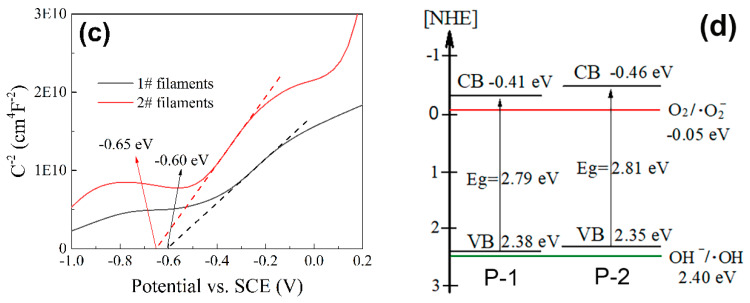
(**a**) UV-visible diffuse reflectance spectra, (**b**) UV photoelectron spectroscopy (UPS) spectra, (**c**) Mott–Schottky (M–S) curves and (**d**) the electronic band structure diagram of the P–1 and P–2 filaments.

Because the PL emission can disclose the charge carrier trapping, migration and recombination processes of semiconductor photocatalysts, the steady-state PL spectra of the P–1 and P–2 filaments at an excitation wavelength of 340 nm are revealed in Figure 5c. It is known that the lower the PL signal, the lower recombination and higher separation efficiency of the electron-hole pairs [64]. The PL intensity of the P–2 filaments is lower than that of the P–1 filaments, suggesting that the recombination of electron-hole pairs on the surface of the P–2 filaments is effectively restrained. So the incorporation of Ag nanoparticles could effectively inhibit the recombination of photo-generated electron-hole pairs of TiO_2_, which is helpful for the photocatalytic behaviors. Another possible reason could be the greater light absorption capability of the P–2 filaments than that of the P–1 filaments (Figure 6a).

To elucidate the photo-physical properties of photo-excited charge carriers, the time-resolved transient PL spectra under excitation at 340 nm is disclosed in Figure 5d. The fitted emission decay data are listed in Table 2 by using the double-exponential formula: *y* = A+*B*_1_e^(−*t*/*τ*1)^ + *B*_2_e^(−*t*/*τ*2)^ [65], where *y* is the emission intensity at any time *t*; *A* is the baseline correction (y-offset); *B*_1_ and *B*_2_ are the constants; *τ_1_* and *τ_2_* are the decay times for the exponential components. The average decay lifetime *τ* can be calculated using the following equation: *τ* = (*B*_1_*τ*_1_^2^ + *B*_2_*τ*_2_^2^)/(*B*_1_*τ*_1_ + *B*_2_*τ*_2_) [65]. The average decay lifetime of the P–1 filaments is reduced from 3.06 ns (χ^2^ = 1.159) to 2.31 ns (χ^2^ = 1.200) when the Ag nanoparticles are incorporated into TiO_2_ nanoparticles. The shortened decay lifetime implies the efficient photo-generated electron transfer [65]. In comparison with the P–1 filaments, the P–2 filaments have a considerably shorter charge lifetime, indicating that there is good photo-generated charge transfer from the conduction band of TiO_2_ to the surface of Ag nanoparticles. Hence, the photocatalytic activity of the dye-sensitized Ag-N co-doped TiO_2_ coated filaments is efficiently improved by creating an interfacial contact between TiO_2_ and Ag nanoparticles.

### 3.4. Optical Properties and Electronic Band Structures

To identify the effects of disperse blue 183 dye and doped Ag nanoparticles on the optical absorption properties of the dye-sensitized Ag-N co-doped TiO_2_ coated PET filaments, the UV-vis diffuse reflectance spectra of the pristine, solely dyed, TiO_2_ coated, Ag-doped TiO_2_ coated, P–1 and P–2 filaments at room temperature along with the optical images are presented in Figure 6a. All the samples exhibit the strong absorption capabilities in UV region mainly due to the π→π* electronic transition in the benzene ring of PET [66]. The absorption performances of pure PET filaments are enhanced after being stained with disperse blue 183 dye and/or coated with TiO_2_ and further improved by the incorporation of Ag nanoparticles. The solely dyed filaments have a strong and broad absorption band from 450 nm to 700 nm because of the characteristic absorption of dye. The absorption edge of the P–1 filaments is red-shifted with respect to the TiO_2_ coated filaments, suggesting that the staining of PET substrate and the doping of N into TiO_2_ can reduce the energy band gap of TiO_2_ coated PET filaments [58]. Additionally, compared with the P–1 filaments, the red-shift of the P–2 filaments slightly increases, which can be attributed to the surface plasmon resonance of Ag nanoparticles (the interference of electromagnetic field with the conduction electrons of Ag nanoparticles embedded in TiO_2_ matrix) [58]. Therefore, the photovoltaic efficiency of the dye-sensitized Ag-N co-doped TiO_2_ coated PET filaments (P–2) is improved by increasing its light absorption in visible light region via dyeing of PET and co-doping of N element and Ag nanoparticles.

The ultraviolet photoelectron spectroscopy (UPS) spectra for both P–1 and P–2 filaments are manifested in Figure 6b. By extrapolating the linear part until their intercepts on the binding energy axis in the UPS spectra, the valence bands can be determined as 2.38 eV for the P–1 filaments and 2.35 eV for the P–2 filaments [67].

The flat band potentials (Fermi levels) are evaluated from the x-intercept of the M–S curves, as noted in Figure 6c. The positive slopes show that both P–1 and P–2 filaments belong to the n-type semiconductor and the multiple space charge regions are existed [68]. By extrapolating 1/C^2^ and potential, the flat band potentials are deduced to be −0.60 eV for the P–1 filaments and −0.65 eV for the P–2 filaments versus the saturated calomel electrode (SCE). The applied potential versus the normal hydrogen electrode (NHE) *E*(vs NHE) can be obtained by converting the potential (vs. SCE) using the following equation: *E*(vs. NHE) = *E*(vs. SCE)+*E*(SCE) [69], where *E*(vs. SCE) is the applied potential versus SCE and *E*(SCE) = 0.24 V at 25 °C. Accordingly, the flat band potentials are equivalent to −0.36 and −0.41 eV versus NHE for the P–1 and P–2 filaments, respectively. Actually, the top potential of the conduction band in most semiconductors is smaller about 0–0.1 eV than the Fermi level and it is set as 0.05 eV here [69]. Thus, the conduction bands are −0.41 and −0.46 eV (Vs. NHE) for the P–1 and P–2 filaments, respectively.

Combined with the UPS results, the band gaps are estimated to be 2.79 eV for the P–1 filaments and 2.81 eV for the P–2 filaments in Figure 6d. Although the P–1 filaments have a slightly narrower band gap than that of the P–2 filaments, the separation efficiency of photo-generated electron-hole pairs of the P–2 filaments is higher than that of the P–1 filaments. It is anticipated that by considering the *E*(O_2_/•O_2_^−^) of −0.05 eV vs. NHE [70], both P–1 and P–2 filaments have more negative potentials to capture O_2_ adsorbed on the surfaces of N-doped TiO_2_ coated filaments to generate •O_2_^−^. However, the photo-generated holes produced by both filaments have not enough potentials to oxide OH^−^ into •OH with regard to *E*(OH^−^/•OH)=2.40 eV (vs. NHE)).

### 3.5. Photocatalytic Performances

The effects of dye-sensitization of N-doped TiO_2_ nanoparticles with disperse blue dyes were investigated [71] after comparing the photocatalytic properties of the PET filaments coated with N-doped TiO_2_ nanoparticles and disperse blue dye-sensitized N-doped TiO_2_ nanoparticles respectively. Also, the effects of Ag-dope on the photocatalytic activities of TiO_2_ nanoparticles were studied [72] after the comparison of the photocatalytic properties of the PET filaments coated with TiO_2_ nanoparticles and Ag-doped TiO_2_ nanoparticles respectively. It was found that the separation efficiency of photo-generated electron–hole pairs of Ag-doped TiO_2_ nanoparticles was improved due to the narrowed band gap and enhanced light absorption capability under visible light irradiation. The reactive radical species including h+, •O_2_^−^ and •OH were involved in the photo-degradation process of organic dyes and h^+^ radicals were the major reactive species.

In this section, we will investigate the influences of dye-sensitization of Ag-N co-doped nanoparticles with disperse blue 183 dye on the photocatalytic properties of PET filaments coated with Ag-N co-doped nanoparticles dye-sensitized and N-doped TiO_2_ nanoparticles dye-sensitized respectively.

**Figure 7:**
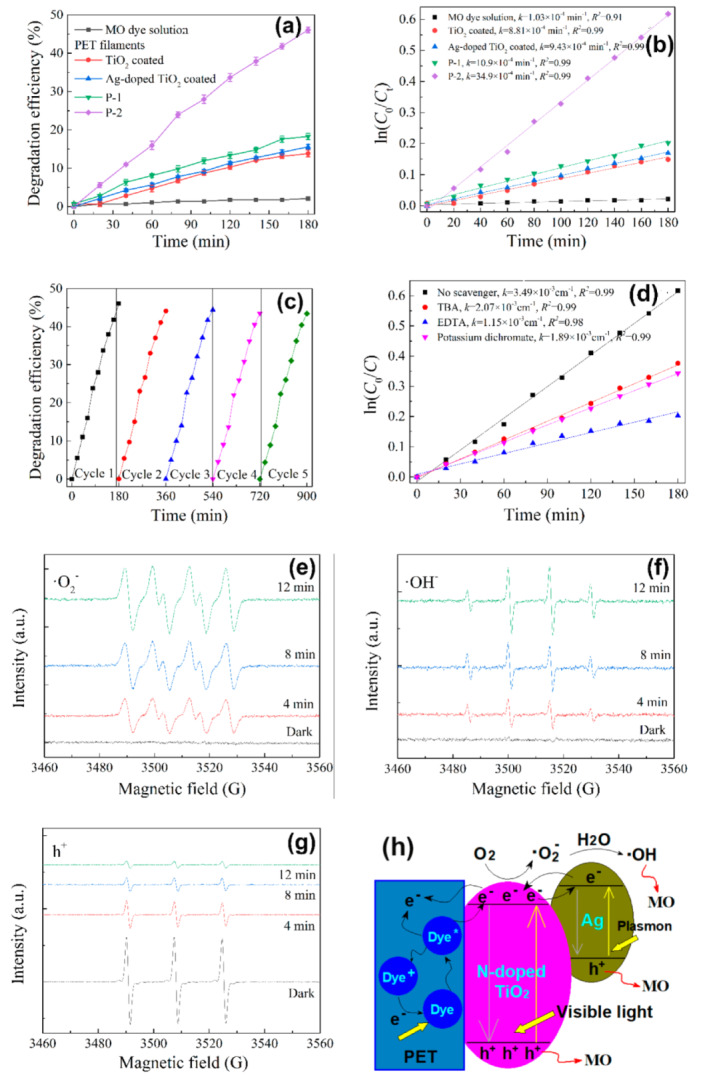
(**a**) Photocatalytic degradation efficiency of methyl orange (MO) dye solution as a function of irradiation time under visible light and (**b**) the corresponding pseudo-first-order kinetic fitting curves for the photo-degradation of MO dye solution; (**c**) cyclic photo-degradation of MO dye solution by the P–2 filaments; (**d**) effects of scavengers on the photo-degradation of MO dye solution by the P–2 filaments; the electron spin resonance (ESR) results for (**e**) •O_2_^-^, (**f**) •OH and (**g**) h^+^ reactive species; (**h**) the proposed visible light photocatalytic mechanism of the P–2 filaments.

The photo-degradation of MO dye solutions by P–1 and P–2 filaments are plotted in Figure 7a,b. It is observed that after 180 min exposure to visible light (Figure 7a), the P–2 filaments possess the highest MO degradation efficiency of 46.1% in comparison to those of the P–1 filaments 18.3%, Ag-doped TiO_2_ coated filaments 15.6% and TiO_2_ coated filaments 13.8%. The degradation efficiency of the P–2 filaments is about 3.33, 2.94 and 2.61 times than those of the TiO_2_ coated, Ag-doped TiO_2_ coated and P–1 filaments, respectively. There is a negligible decrease in the photocatalytic efficiency of the blank MO dye solution. Noticeably, the degradation efficiency of the P–1 filaments is greater than those of both PET filaments coated with TiO_2_ nanoparticles and PET filaments coated with Ag-doped TiO_2_ nanoparticles. This is ascribed to the fact that besides the relative higher loading of TiO_2_ (EDS analysis), the photocatalytic activity of the TiO_2_ coated PET filaments is enhanced by the staining of the PET substrate, which can promote the transfer of electrons from TiO_2_ to PET filaments. The attapulgite–TiO_2_–Fe_x_O_y_ composite was used for the photo-degradation of MO dye solution at ambient temperature, which was irradiated by a 500 W Xe lamp with a 420 nm cutoff filter. 0.25 g of the composite was added into 250 mL of 20 mg/L MO solution containing 1.0 mL of 30% H_2_O_2_. The degradation efficiency was 94.13% after 180 min of irradiation [73]. The TiO_2_ nanohybrids modified by Nb_2_O_5_, SnO_2_, reduced graphene oxide (RGO) and ceramic (SiO_2_/Fe_3_O_4_/ZrO_2_) were prepared for the photo-degradation of MO dye solution at 30 °C to 35 °C. A 300 W Xe lamp was used as the light source with a UV cutoff (λ *>* 400 nm) filter. 3 mg of photocatalyst was dispersed in a 150 mL solution of 20 mg/L MO dye solution and the pH of initial dye solutions was neutral. After 120 min of light illumination, the degradation efficiencies were 95%, 91%, 87%, 81%, 65%, 54% and 23% for the TiO_2_/Nb_2_O_5_/SnO_2_/RGO, TiO_2_/Ceramic/SnO_2_/RGO, TiO_2_/Nb_2_O_5_/SnO_2_, TiO_2_/Ceramic/SnO_2_, TiO_2_/Nb_2_O_5_, TiO_2_/Ceramic and TiO_2_, respectively [74]. The photocatalytic activities of the above two photocatalysts are higher than that of the P–2 filaments mainly because of the stronger irradiation intensity. Moreover, in our previous study, about 1.0 g of TiO_2_ modified and dyed PET fabrics was immersed in 50 mL of 5 mg/L MO dye solution exposed to a 500 W Xeon lamp. A 400 nm cut-off UV filter was used to simulate the visible light irradiation. After 6 h of irradiation at 10 °C, the photocatalytic efficiency was 31% [48], smaller than 46% of this study. To quantitatively identify the reaction kinetics of MO solution degradation, the experimental data were fitted using the Langmuir–Hinshelwood model [75], as expressed by the formula: *ln*(*C*_0_/*C_t_*) = *kt*, where *k* is the pseudo-first-order apparent rate constant (min^−1^), *C*_0_ and *C_t_* are the MO solution concentration at the initial time and time *t*, respectively. From the linear fitting curves of *ln*(C_0_/C_t_) vs. irradiation time t (Figure 7b), the MO degradation efficiency constant k values are calculated to be 1.03 × 10^−^^4^, 8.81 × 10^−^^4^, 9.43 × 10^−^^4^, 10.90 × 10^−^^4^ and 34.90 × 10^−^^4^ min^−1^ for the blank MO solution, TiO_2_ coated, Ag-doped TiO_2_ coated, P–1 and P–2 filaments, respectively. Obviously, the P–2 filaments have the largest k value, which is 4.0, 3.7 and 3.2 times higher than those of the TiO_2_ coated, Ag-doped TiO_2_ coated and P–1 filaments. Therefore, the synergistic enhancement of the Ag-N co-doping [76] and dyeing of PET can significantly improve the photocatalytic activity of the TiO_2_ coated PET filaments.

It is deduced from Figure 7c that after five successive cycles of MO photo-degradation under 180 min visible light irradiation, the degradation efficiency of the P–2 filaments can be maintained to a considerable degree, implying the good photocatalytic stability and reusability. The results also verify that the MO solution degradation is photocatalytic reaction rather than photo-corrosive one [77].

To further elucidate the photocatalytic redox process of the photo-degradation of MO dye, the trapping experiments shown by Figure 7d are conducted to identify the reactive radical species of •OH, h^+^ and •O_2_^−^ by adding 0.5 mL TBA, 1 mM EDTA and 0.5 mM potassium dichromate in the MO dye solution, respectively. It is apparent that the photodegradation of MO solution is slightly inhibited by the presence of TBA or potassium dichromate but seriously inhibited by the addition of hole scavenger EDTA as compared to that without addition of quencher. This indicates that the active holes are the major reactive radicals and •OH and •O_2_^−^ also play certain roles in the photodegradation process of MO solution.

The ESR spectra of •OH, •O_2_^−^ and h^+^ of the P–2 filaments are exhibited in Figure 7e–g. It is clear that there are no signals in the ESR spectra of •OH and •O_2_^−^ radicals in the dark. By exposure to visible light, the peak intensities of both •OH and •O_2_^−^ radicals increase gradually with the increase of irradiation time, confirming that certain amounts of •OH and •O_2_^−^ radicals are formed in the dye-sensitized Ag-N co-doped TiO_2_ coated PET filaments (Figure 7e,f). However, an opposite rule is observed in the ESR spectra of h^+^ radicals (Figure 7g). A weaken signal of h^+^ radicals (equal to strong e^-^ radicals) is detected in dark owing to the electrophilicity of TEMPO [78]. As the irradiation time is prolonged, the peak intensity of e^-^ radicals is drastically reduced, suggesting that a large number of h^+^ radicals are generated in the P–2 filaments under visible light irradiation. Hence, the ESR measurements are consistent with the results of trapping experiments.

### 3.6. Photocatalytic Mechanism Analysis

According to the aforementioned experimental results, the possible mechanism for the enhanced photocatalytic activity of the P–2 filaments is outlined in Figure 7h. The photo-generation of electron–hole pairs would abide the following steps.

Step (i), under visible light illumination, the molecules of water insoluble disperse blue dye embedded into PET filaments would be excited.

Step (ii), a few excited electrons would transfer from the dyed PET substrate to the Ag-N co-doped TiO_2_ nanoparticles which are contacted with the dyed PET substrate. Also, the other excited electrons would be trapped by the PET substrate because of the large electronegativity of PET (6.84 eV) relative to TiO_2_ (5.81 eV) according to the Mulliken electronegativity theory [79].

Step (iii), it has been demonstrated that the band gap of anatase TiO_2_ can be narrowed by doping of substitutional and interstitial N impurities to a certain extent [80,81]. Thus the Ag-N co-doped TiO_2_ nanoparticles could be excited by visible light irradiation due to the formation of localized occupied states in the band gap of TiO_2_ [82].

Step (iv), some photo-induced electrons produced by the Ag-N co-doped TiO_2_ nanoparticles would migrate from the coating of Ag-N co-doped TiO_2_ nanoparticles to the dyed PET filaments owing to the relatively large electronegativity of PET. Meanwhile, the Ag nanoparticles engaged with the N-doped TiO_2_ nanoparticles have the capability to capture the photo-induced electrons [57]. The incorporated Ag nanoparticles would act as the effective electron scavenger to trap the conduction-band electrons of TiO_2_, which not only facilitate the separation of electrons and holes but also suppress the recombination of photo-generated charge species and thereby prolong the lifetime and mobility of the charge carriers.

Step (v), on the other hand, the incorporated Ag nanoparticles can absorb the photons via the localized surface plasmon resonance to generate high energy electrons and these electrons have a tendency to transfer to the N-doped TiO_2_ nanoparticles [83] and might further transfer to the dyed PET substrate. This is caused by the interfacial charge-transfer transitions between PET polymer molecules and N-doped TiO_2_ nanoparticles without loss of energy [84].

Step (vi), consequently, the photo-induced electrons generated by the Ag-N co-doped TiO_2_ nanoparticles are trapped by the dyed PET filaments.

The presence of water insoluble disperse dye in PET polymer plays an important role in the separation of photo-generated electron–hole pairs. The PET polymer and water insoluble disperse dye can improve the separation efficiency of photo-generated electrons and holes, resulting in the enhanced photocatalytic activity of the dye-sensitized Ag-N co-doped TiO_2_ coated PET filaments. By considering the electronic band structures (Figure 5d), the predominant reactive radical species for the MO dye photo-degradation by the dye-sensitized Ag-N co-doped TiO_2_ coated PET filaments are the photo-generated holes. The hydroxyl and superoxide radicals play a minor role to photocatalytic reaction.

## 4. Conclusions

In this paper, the enhanced photocatalytic activity of a composite photocatalyst composed of PET filaments loaded with Ag-N co-doped TiO_2_ nanoparticles sensitized with water-insoluble disperse blue 183 dye, which was formed in a facile one-step hydrothermal process, under visible light irradiation was achieved. The synergetic mechanism of both metal-nonmetal co-doping and dye sensitization on the photocatalytic activities of TiO_2_ nanoparticles under visible light irradiation was investigated.

The microstructure and photocatalytic properties of the as-synthesized TiO_2_ nanocomposites as well as the as-prepared PET filaments were systematically characterized. FESEM, XRD, TEM and XPS results indicated that the Ag-N co-doped and dye-sensitized anatase TiO_2_ nanoparticles were chemically grafted on the PET filament surface via the O-Ti^3+^/O-Ti^4+^ bonds. Diffuse reflectance spectra (DRS), UPS and M–S results showed that the absorption edge of the dye-sensitized PET-Ag-N-TiO_2_ composite photocatalyst was red-shifted because of the surface plasmon resonance of Ag nanoparticles in comparison with the PET filaments loaded with N-doped TiO_2_ nanoparticles sensitized with water-insoluble disperse blue dye. The valence bands and conduction bands were 2.38 eV and −0.41 eV for the dye-sensitized N-doped TiO_2_ coated PET filaments, which was slightly greater than 2.35 eV and −0.46 eV for the dye-sensitized Ag-N co-doped TiO_2_ coated PET filaments, respectively. The superior photocatalytic activity of the dye-sensitized PET-Ag-N-TiO_2_ composite photocatalyst was primarily ascribed to the quick separation of photo-generated electron-hole pairs and efficient interfacial charge transfer, which were confirmed by the results of *i*–*t*, EIS, steady-state PL and time-resolved transient PL measurements.

The photocatalytic performance of the PET filaments coated with the dye-sensitized, Ag-N co-doped TiO_2_ nanoparticles was evaluated via its capacity of photo-degrading MO dye under visible light irradiation. The results of photo-degradation of MO dye solution and trapping experiments as well as ESR measurements showed that the holes, in comparison with •OH and •O_2−_ radicals, were the main reactive radical species for the PET filaments coated with dye-sensitized Ag-N co-doped TiO_2_ in the photo-degradation of MO dye under visible-light irradiation. PET substrate and water insoluble disperse blue dye made great contribution to the superior photocatalytic activity of the dye-sensitized Ag-N co-doped TiO_2_ coated PET filaments, which not only generated more holes but also inhibited the recombination of electron-hole pairs by the transferal of electrons to the dyed PET substrate and Ag nanoparticles. In comparison with the PET filaments coated with TiO_2_ nanoparticles, the PET filaments coated with Ag-N co-doped and dye-sensitized TiO_2_ nanoparticles exhibited great enhanced light absorption capacity, efficient separation efficiency of electron-hole pairs and substantial photocatalytic activity in degradation of MO dye under visible-light irradiation. The resultant photocatalytic composite filaments were evident to be capable of repeatedly photo-degrading MO dye without losing its photocatalytic activity significantly. Thus the proposed photocatalytic composite structure could be taken as a novel approach to design textile materials based composite photocatalysts for the photo-degradation of organic pollutants.

## Figures and Tables

**Table 1:** The results of X-ray photoelectron spectroscopy (XPS) elemental analysis of the P–1, P–2, TiO_2_ coated and Ag-doped TiO_2_ coated filaments.

PET Filaments	Peak	Binding Energy (eV)	FWHM (eV)	Area (Cps•eV)	Relative Sensitivity Factor (RSF)	Atomic Concentration (%)
P–1	C_1s_	285.3	5.168	5124.6	0.278	73.02
	N_1s_	396.7	0.242	91.5	0.477	0.74
	O_1s_	532.2	4.409	5227.8	0.780	24.43
	Ti_2p_	459.0	3.170	957.4	2.001	1.81
P–2	C_1s_	282.6	2.421	6575.5	0.278	72.97
	N_1s_	396.1	0.313	75.1	0.477	0.47
	O_1s_	529.6	4.167	6577.4	0.780	23.95
	Ti_2p_	456.3	2.196	1689.3	2.001	2.49
	Ag_3d_	365.6	0.386	242.4	5.987	0.12
TiO_2_ coated	C_1s_	287.0	1.924	770.8	0.278	75.02
	O_1s_	532.0	2.134	750.2	0.780	23.95
	Ti_2p_	460.1	1.795	79.4	2.001	1.03
Ag-doped TiO_2_ coated	C_1s_	287.2	2.020	695.4	0.278	81.82
	O_1s_	531.8	2.187	417.9	0.780	16.13
	Ti_2p_	459.8	1.795	111.2	2.001	1.73
	Ag_3d_	367.2	1.410	58.6	5.987	0.32

**Table 2:** Fitting parameters of the time-resolved PL data using two-exponential decay kinetics.

PET Filaments	τ_1_ (ns)	B_1_ (%)	τ_2_ (ns)	B_2_ (%)	τ_average_ (ns)	χ^2^
P–1	0.96	75.19%	9.01	24.81%	3.06	1.159
P–2	0.87	72.90%	6.66	27.10%	2.31	1.200
